# Photocatalytic Activity: Experimental Features to Report in Heterogeneous Photocatalysis

**DOI:** 10.3390/ma11101990

**Published:** 2018-10-15

**Authors:** Md. Ariful Hoque, Marcelo I. Guzman

**Affiliations:** 1 Department of Chemistry, University of Kentucky, Lexington, KY 40506, USA; md.hoque@uky.edu; 2 Center for Applied Energy Research, University of Kentucky, Lexington, KY 40511, USA

**Keywords:** heterogeneous photocatalysis, photocatalytic activity, photocatalytic efficiency, rate per weight, apparent quantum efficiency

## Abstract

Heterogeneous photocatalysis is a prominent area of research with major applications in solar energy conversion, air pollution mitigation, and removal of contaminants from water. A large number of scientific papers related to the photocatalysis field and its environmental applications are published in different journals specializing in materials and nanomaterials. However, many problems exist in the conception of papers by authors unfamiliar with standard characterization methods of photocatalysts as well as with the procedures needed to determine photocatalytic activities based on the determination of “apparent quantum efficiencies” within a wavelength interval or “apparent quantum yields” in the case of using monochromatic light. In this regard, an astonishing number of recent research articles include claims of highly efficient (photo)catalysts or similar terms about materials with superior or enhanced efficiency for a given reaction without proper experimental support. Consequently, the comparison of the efficiencies of photocatalysts may result as being meaningless, especially when reports are only based on expressions determining (1) a reaction rate per weight of catalyst or its surface area, (2) quantum efficiencies or quantum yields, and (3) turnover frequencies or turnover numbers. Herein, we summarize the standards needed for reporting valuable data in photocatalysis and highlight some common discrepancies found in the literature. This work should inform researchers interested in reporting photocatalysis projects about the correct procedures for collecting experimental data and properly characterizing the materials by providing examples and key supporting literature.

## 1. Introduction

The use of photocatalysis has attracted a great deal of attention in the nanomaterials community to solve problems related to the mitigation of air pollution and environmental remediation of contaminated waters (i.e., the degradation of dyes) [1]. More recently, photocatalysis has been implemented for the conversion from solar to chemical energy. For example, the photocatalytic reduction of carbon dioxide (CO_2_) into carbon monoxide (CO) and organic compounds with one (e.g., CH_3_OH, HCOOH, and CH_4_) and two carbon atoms (e.g., HOOCCOOH, HCOCOOH, and CH_3_CH_2_OH) is of interest for the storage of solar energy in chemical bonds [2,3,4,5,6]. Similarly, the power of photocatalytic water splitting for producing molecular hydrogen (H_2_) and oxygen (O_2_) can also serve to produce chemical fuel [7,8]. Thus, the synthesis of advanced semiconductor materials with valuable photocatalytic properties is continuously generating new catalysts for driving a variety of reactions more efficiently [9,10]. The produced new catalysts are often tested against a specific reaction such as the degradation kinetics of a dye, resulting in works that claim to have improved the photocatalytic efficiency (or enhanced the activity) of the process relative to previous materials, modified stoichiometric ratios, variable geometries, or single components integrated into nanocomposite heterostructures [11,12]. However, such measurements are insufficient to report photocatalytic activities because they lack a mechanism to account for the number of photons driving the process [13,14].

In addition, a trained expert in photocatalysis should find it concerning that a significant number of articles could be published with poorly characterized materials [15]. The common doubly problematic case corresponds to articles that neither explain the experimental details, conditions, and procedures followed when synthesizing and characterizing the materials, nor consider appropriate protocols for reporting measurements of photocatalytic activity. Various practices can be found in the literature which have been applied for comparing the activity or reactivity of the photocatalysts, which include reporting (1) the rate of formation of a product or loss of a reactant per gram (or alternatively per surface area) of catalyst, and (2) the turnover numbers (or the turnover frequencies) [13]. However, as mentioned above, the principal concern in a reaction promoted by light is to account for the numbers of photons driving it and not the amount of photocatalyst participating in the process. Therefore, attempts to report the activity of photocatalysts in any reaction that omit computing the number of photons cannot be justified and are strongly discouraged. Several special considerations must be taken into account for comparing the efficiency of the photocatalysts. These experimental features are needed to report and compare the activity of photocatalysts in terms of apparent quantum efficiencies (AQE) or apparent quantum yield (AQY), not simply on reaction rates, are presented below. Other important outlines will be briefly addressed to guide nonexperts to correctly determine photocatalytic performances.

**Figure 1:**
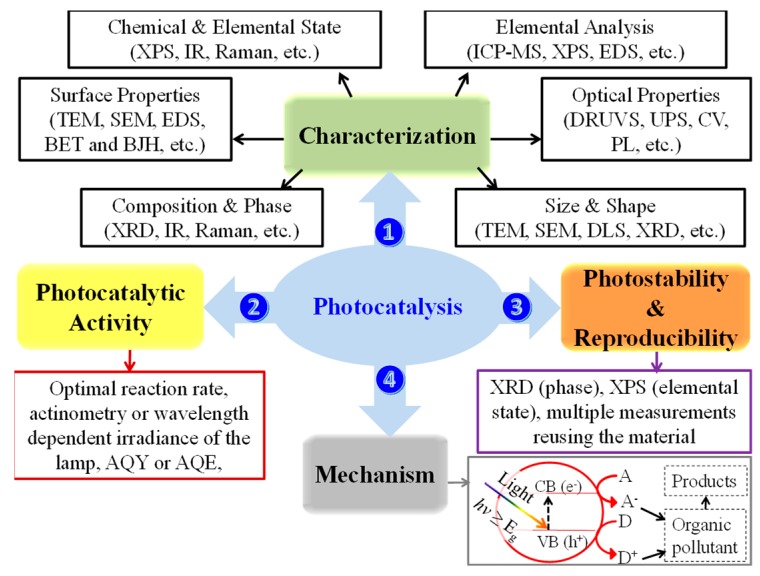
Representation of the four features to report in heterogeneous photocatalysis: (1) material characterization, (2) evaluation of photocatalytic activity, (3) determination of the photocatalyst stability and data reproducibility, and (4) proposal of a mechanism. Key for abbreviations: A, electron acceptor; AQE, apparent quantum efficiency; AQY, apparent quantum yield; BET, Brunauer−Emmett−Teller; BJH, Barrett-Joyner-Halend; CB, conduction band; CV, cyclic voltammetry; D, electron donor; DLS, dynamic light scattering; DRUVS, diffuse reflectance UV-visible spectroscopy; EDS, energy dispersive X-ray spectroscopy; *E*_g_, bandgap energy; *e*^−^, electron; *h*^+^, hole; *hν*, photon energy; ICP-MS, inductively coupled plasma mass spectrometry; IR, infrared spectroscopy; PL, photoluminescence spectroscopy; SEM, scanning electron microscopy; TEM, transmission electron microscopy; UPS, ultraviolet photoelectron spectroscopy; VB, valence band; XPS, X-ray photoelectron spectroscopy; and XRD, X-ray diffraction.

Figure 1 summarizes the four critical features that need to be reported in a photocatalysis paper that are described in the next sections: (1) the proper characterization of materials, (2) the evaluation of photocatalytic activity, (3) the determination of the photocatalyst stability and data reproducibility, and (4) proposal of a meaningful mechanism.

## 2. Reporting the Thorough Characterization of Materials

The characterization of the photocatalyst should always aim to include (1) the composition of the material (with the concentration of any host and dopant components), (2) its optical properties, (3) the size and shape of the particles, and (4) measurements of the surface properties [15,16]. Providing this fundamental information will allow the full and proper evaluation of the photocatalyst. The characterization of the crystalline or amorphous materials must include the X-ray diffraction (XRD) pattern, typically collected in a diffractometer for powder samples or thin films. The diffractogram can provide information of the composition of the nanomaterials (and the size of crystallites), the identification of phases, and the presence of impurities. Reporting the crystalline behavior (amorphous or crystalline) of the materials and the exact crystalline phase (some photocatalysts can have more than one crystal structure) is an important task because each phase usually displays different photocatalytic activity. Infrared (IR) and Raman spectroscopies can also provide evidence to supplement the knowledge gained from XRD.

Accepted methodologies to report the size and shape of the particles are the use of micrographs collected by transmission electron microscopy (TEM) or scanning electron microscopy (SEM), which can both typically be combined with the use of energy-dispersive X-ray spectroscopy (EDS) to reveal the elemental composition of the surface. Dynamic light scattering (DLS) measurements can also contribute information to characterize the size distribution histogram of various particles suspended in a liquid that more realistically represents the aggregates participating in photocatalytic processes with colloids. In addition, the bulk elemental analysis for host and/or dopant species requires the characterization employing either inductively coupled plasma (ICP) with mass spectrometry (MS) or atomic emission spectroscopy (AES) detection, or even more labor-intensive atomic absorption spectroscopy (AAS) if the previous methods are not available. The use of X-ray photoelectron spectroscopy (XPS) is also highly recommended for quantitatively reporting the elemental composition of the surface of the material as well as the chemical and electronic states of the elements in the material. The characterization of specific surface areas and pore size distribution is typically performed by the Brunauer−Emmett−Teller (BET) method and Barrett-Joyner-Halend (BJH) method from the adsorption–desorption isotherms of a gas such as N_2_ or CO_2_.

The characterization of the optical properties of the nanomaterials requires the use of diffuse reflectance UV–visible spectroscopy (DRUVS) to find the optical bandgap (or band gap energy) of semiconductors from a Tauc plot [17]. In the Tauc plot, the Kubelka–Munk function, $F\left\{R_{\infty }\left(\lambda \right)\right\}$, is proportional to the absorption coefficient of the material (*α*) as it varies according to the expression (*αhν*)^1/*n*^, which is plotted against the energy of the photons (*hν*), where α is the absorption coefficient, *h* is Planck’s constant, and *ν* is the frequency of light. After carefully fitting the best line to the previous plot, an extrapolation to zero shows the intercept corresponding to the optical bandgap [18]. The work should consider the exponent (*n*) for the bandgap of the semiconductor material, which can correspond to an allowed direct (*n* = 0.5), allowed indirect (*n* = 2), forbidden direct (*n* = 1.5), or forbidden indirect (*n* = 3) transition. In addition, some researchers determine the electrochemical bandgap using cyclic voltammetry (CV) measurements [19].

Ultraviolet photoelectron spectroscopy (UPS) should be used to determine the work function of the photocatalyst. The construction of the energy band diagram of pure materials (before contact) and composites (after contact) enabled by UPS data is critical to determine the position of the valence band maximum. To calculate the conduction band minimum potential, the previously determined bandgap is needed. Experimentally, the full width of the UPS spectrum is measured and subtracted from the photon energy of the exciting radiation to obtain the difference that is called the work function [20]. An example for the use of UPS for characterizing a Cu_2_O/TiO_2_ heterojunction is provided in the work of Aguirre et al. [21]. Finally, photoluminescence measurements can serve for the evaluation of the recombination rate of the photogenerated electron–hole pairs [22].

**Figure 2:**
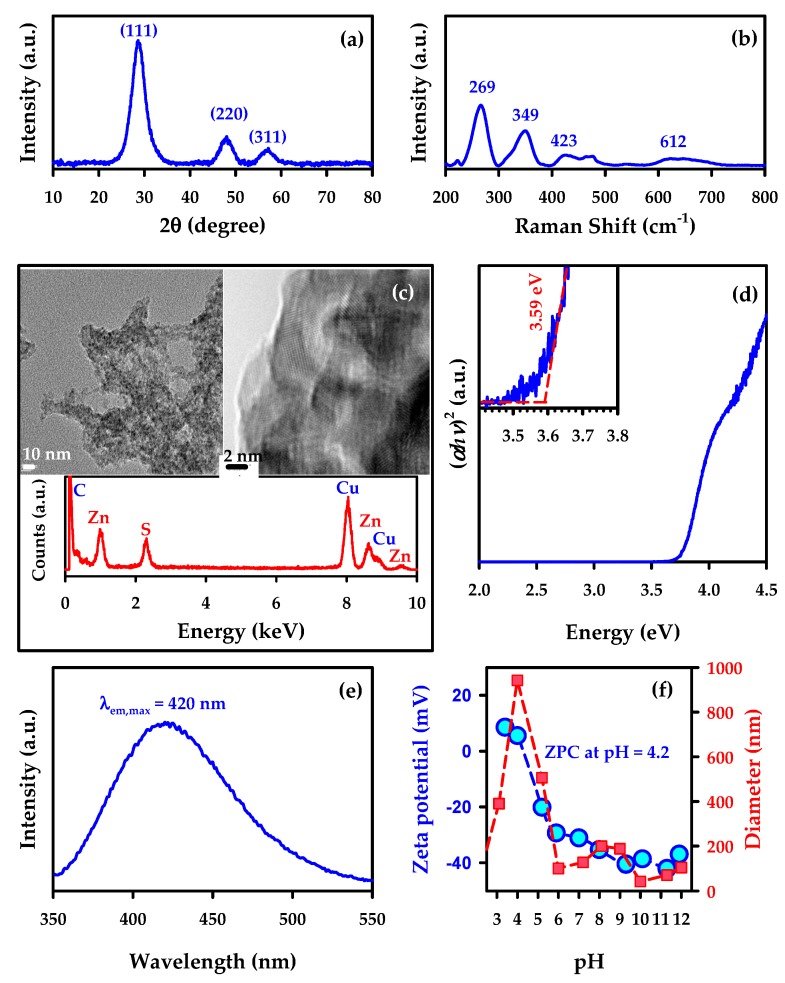
Example for the characterization of a ZnS model semiconductor. Further experimental details about the synthesis of ZnS, data collection, and interpretation can be found in references [2,9]. (**a**) Powder X-ray diffractogram (XRD) for cubic ZnS. (**b**) Raman spectrum for cubic ZnS. (**c**) Transmission electron microscopy (TEM) of ZnS with energy-dispersive X-ray spectroscopy (EDS), showing that the 1:1 stoichiometric ratio of Zn:S is maintained on the surface. (**d**) Tauc plot obtained by plotting the square of the product of the absorption coefficient (proportional to the Kubelka–Munk function recorded by DRUVS) and energy in eV vs energy. A linear extrapolation provides the bandgap of 3.59 eV (or *λ* = 345 nm) for colloidal ZnS suspended in water. (**e**) Photoluminescence spectrum of colloidal ZnS suspended in water and excited at *λ* = 300 nm with an emission maximum at *λ*_em,max_ = 420 nm. (**f**) Dynamic light scattering (DLS) analysis of colloidal ZnS suspended in water showing the (blue circle) zeta-potential and (red square) particle size (diameter from the unimodal distribution) at variable pH with a zero point of charge (ZPC) at pH = 4.2.

In order to illustrate the utility of several techniques, Figure 2 displays the characterization of zinc sulfide (ZnS) as a simple model photocatalyst [2,9]. Figure 2 includes a powder XRD diffractogram showing the composition of the cubic crystallites (which also allows evaluating the size of the crystallites), a Raman spectrum that provides additional support to the cubic phase of ZnS, a TEM–EDS reporting the surface features and composition of photocatalyst mounted on a carbon coated copper grid, a Tauc plot from the DRUVS spectrum of the direct semiconductor with an allowed transition corresponding to a bandgap of 3.59 eV for the colloids suspended in water, a photoluminescence spectrum for a suspension of the material under excitation wavelength of 300 nm, and the particle size distribution and zeta potential of the colloidal material suspended in water at variable pH.

## 3. Evaluation of Photocatalytic Performance

**Figure 3:**
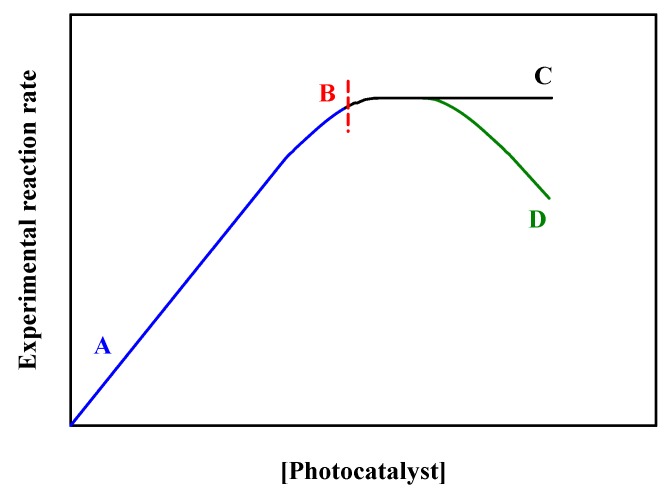
Experimental rate of the photocatalyzed reaction vs the concentration of photocatalyst.

A simple way to start the photocatalysis work with colloidal suspensions is to study the dependence of photoreaction rate with increasing catalyst concentration. It is well known that the rate of photoreaction both in homogenous [23] and heterogeneous [24] systems should initially increase linearly with catalyst concentration, as depicted from region A to B in Figure 3. This initial linear behavior is due to the larger absorbed photon flux for increasing [photocatalyst]. When transitioning from region B to C in Figure 3, the maximum rate is reached and stays constant in the concentration interval represented, which corresponds to the optimal light absorption. In some cases, the photocatalytic rate may decrease, as displayed for the transition from region B to D, due to the increased scattering and reduced penetration depth of the incident light [25]. The key point here is that if a photocatalytic reaction is studied in the linear interval A–B (Figure 3), at least one extra point in the plateau region B–C should be recorded to ensure the behavior of the system is known under optimal light absorption conditions. In other words, working under optimal light absorption conditions significantly simplifies the interpretation of the photocatalytic mechanism to allow the comparison of quantum yields, which are meaningful representations of photocatalytic reaction rates [26,27] based on the number of absorbed photons.

In opposition to the proportionality existing between the rate of a thermal heterogeneous catalysis reaction and the number of active sites of the catalysts, the photocatalytic rate constant is not controlled by the number of active sites (or the amount of catalysts) [26]. Therefore, reporting and comparing any sort of efficiency of the photocatalytic process exclusively based on the reaction rate per weight (or number of active sites) of catalysts is not recommended. Such comparison of reactions rates per weight (or number of active sites) of catalysts is specific to each material and photoreactor (i.e., the geometry) system. In other words, changing the photoreactor (different design), the weight of the catalysts used, or the type of materials alters the reaction rate. The use of reaction rates per weight or even reaction rates per surface area of catalyst cannot reflect the intrinsic photocatalytic activity. Instead, it is important to recall that the optimal reaction rate (in the plateau region of Figure 3) must be reported. This is typically a key condition for comparing the photocatalytic behavior of colloidal suspensions. Nevertheless, in some photocatalytic reactions, surface area is an important parameter to report if the rate is limited by the adsorption of reactant species to the catalytic surface.

A typical photocatalysis experiment also allows for an adsorption/desorption equilibrium to be reached in the dark (before starting irradiation with a prewarmed lamp). Such a simple precaution should ensure working under the optimal reaction rate for that loading (or per unit surface area) of photocatalyst. There is also a relationship between photocatalytic reaction rate data to the charge separation efficiency (CSE) induced in the semiconductor surface by the different surface area and the particle size of the catalysts. The surface area itself is not a direct measurement of CSE and hence of photocatalytic activity [13]. Therefore, it is crucial to discuss how the photocatalytic activity should be reported and not just to normalize reaction rates by weight, surface area, or number of active sites.

The primary concern in a photochemical process is to account for the number of photons that drive the reaction of interest. The correct measurement of either the number of photons or their energy striking into a geometrical surface area of the photoreactor (covered by the photocatalyst) is needed to determine the photocatalytic efficiency. For the photochemical reaction (1),

(1) R+hν→P

the rate of transformation of the reactant *R* into the product *P* is equal to the product of the quantum yield of reactant loss (Φ(*R*)*_λ_*) at the irradiation wavelength (*λ*) (or quantum efficiency for an interval Δ*λ*) and the absorbed photon flux (*I_a_*, i.e., the number of photons absorbed at a given of wavelength per second and per unit volume) as given by Equation (2):

(2) $$-d\left[R\right]/dt=\Phi {\left(R\right)}_{\lambda }\;I_{a}$$

where *I_a_* depends on the light path (*d*), the extinction/absorption coefficient (*κ*) of the photocatalysts and substrate, and the incident photon flux (*I*_0_) as shown in Equation (3):

(3) $$I_{a}=\kappa \;d\;I_{0}$$

The photochemical rate constant (*k*) depends on the quantum yield, the absorbed photon flux, and the concentration of substrate [R] as presented in Equation (4) [26]:

(4) $$k=\left(\Phi {\left(R\right)}_{\lambda }\;\kappa \;d\;I_{0}\right)/\left[R\right]$$

Because the key driver of the photocatalytic process is the absorption of photons, the quantum yield (not just the rate constant) must be used for reporting and comparing photocatalytic activities [26]. However, researchers from different fields may have introduced different terms for reporting the photocatalytic activity, obtained by dividing the number of products formed or reactants lost with the number of photons absorbed by the photocatalysts in a narrow or broad wavelength range. The use of the words “quantum efficiency” refers to the case of irradiation within a broad wavelength range (Φ(*R*)_Δ*λ*_). Instead, the use of “quantum yield” (Φ(*R*)*_λ_*) refers to the use of monochromatic radiation (or constrained to a very narrow range of wavelengths). The two previous expressions are defined in a report by IUPAC to account for the number of absorbed photons [28]. Also related is the proposal of the International Standard Organization (ISO) for a standard test protocol (e.g., ISO 22197-1, a standard test method for nitric oxide (NO) oxidation [29]) to compare photocatalytic performance with other photocatalysts. A major challenge in heterogeneous photocatalysis is to determine the number of absorbed photons when working with a powder suspension or thin film [30]. This complication for determining the absorption of light (i.e., by the suspended particles) is caused by losses due to scattering and reflection of light, that are in practical terms difficult to correct [31]. Thus, the value of the pure terms “quantum efficiency” and “quantum yield” in heterogeneous photocatalysis is limited by the fact that the number of incident but not absorbed photons is generally associated to a considerable level of uncertainty. For this reason, IUPAC actually recommends to rather use the terms “apparent quantum yield” (AQY) or “apparent quantum efficiency” (AQE) [28], which correspond to lower limits of the quantum yield and quantum efficiency, respectively.

A common mistake to avoid is the comparison of reaction rates assuming the absorbed light intensity should be same in different reactions. Light absorption may be altered even by a slight change of an experimental condition (i.e., in the design of the irradiation setup, the nature of suspension, etc.). Typically, the measurement of the incident photon flux on the preferably flat optical window of the photoreactor is recorded [30,32,33,34] with a calibrated spectral radiometer. For example, the number of incident photons can be easily measured using a photodiode. Sometimes, such a determination is performed by chemical actinometry. For example, measurements of the incident photon flux for broader wavelength ranges can be performed by potassium ferrioxalate actinometry, for which quantum efficiency is reported to be constant within the values Φ = 1.25–0.9 for the wavelength range from 250 to 500 nm [28,35]. Special care must be taken with this very sensitive actinometer to work in a dark room. In addition, the use of the ferrioxalate actinometer has been effective to normalize the output of a calibrated air-cooled thermopile detector [2], which can be employed to measure the light intensity of individual experiments with high-intensity lamps. It is unusual for the number of absorbed photons for the same number of incident photons to remain constant due to the variable fraction of reflected and scattered photons among experiments or different materials. Thus, comparisons using only *I*_0_ (instead of *I_a_*) are not reliable and prevent the researcher from comparing their results to those from other groups [26].

The determination of AQE can be alternatively performed with diffuse reflectance measurements of the semiconductor for a material that is the only light-absorbing species [26]. In this approach, *κ* in Equation (3) can be substituted by the product of $F\left\{R_{\infty }\left(\lambda \right)\right\}$ and the scattering coefficient, *S_λ_* (usually assumed to be a constant) [36,37], to obtain the corrected rate of photon absorption, as indicated below:

(5) $$I_{a}=F\left\{R_{\infty }\left(\lambda \right)\right\}\;S_{\lambda }\;I_{0}$$

Such a correction must be applied with the understanding that it is valid only if scattering and reflection remain the same for both DRUVS measurements and photocatalytic experiments. This is the case when the surface properties in the experiments or photoreactors to be compared are the same. Reporting AQE for photocatalyzed reactions by combining measurements of reaction rates and diffuse reflectance spectra based on ISO standards is especially useful for systems studying reactions on the solid/gas interface. Finally, if monochromatic light is used during the photocatalytic process, it is technically much simpler to report the AQY.

Two final concepts of general interest regarding photoelectrochemical studies are the internal quantum efficiency and the external quantum efficiency [38]. The internal quantum efficiency refers to the absorbed photon to current efficiency, i.e., the photocurrent collected per absorbed photon flux as a function of the irradiation wavelength. The external quantum efficiency is also called the incident photon to current efficiency, i.e., the photocurrent collected per incident photon flux as a function of the irradiation wavelength [38].

## 4. Photocatalyst Stability and Data Reproducibility

In addition to reporting the AQE as discussed above, it is important to consider the stability of the photocatalyst during irradiation for a long time. Extended irradiation can cause a decrease in the photocatalytic performance because of the deactivation of the photocatalysts, reoxidization of the reduction products, and poisoning effects [39]. The adsorption of poisoning species on the reactive sites of the catalyst surface can deactivate the photocatalyst. However, the most serious case of photocatalytic deactivation is produced by the oxidation and/or photocorrosion of the materials [21,39]. Thus, the stability of the photocatalysts under the reaction conditions should be considered as a key aspect for improving photocatalytic efficiency. It is good practice to include data from several characterization techniques (i.e., XRD for phase analysis, XPS for chemical state, electron microscopy for surface analysis with particle size, etc.) for the catalysts after photoirradiation that contribute evidence of the catalyst’s stability. In addition, appropriate datasets showing the reproducibly of the results (i.e., using the same material in two or more cycles) should be provided to demonstrate the reusability and durability of the photocatalysts [15,21]. Of course, the reproducibility of the experimental data and the statistical assessment of such are important aspects of any study of photocatalytic activity too [13]. As in any physical science, demonstrating the reproducibility of the photocatalysis work is the key for building trust in the results reported [13]. Photocatalysis work should show the statistics of experiments that have been performed many times and include error bars. Similarly important is to provide as much detailed and clear information as possible for others to be able to reproduce the work from the beginning to the end [40].

**Figure 4:**
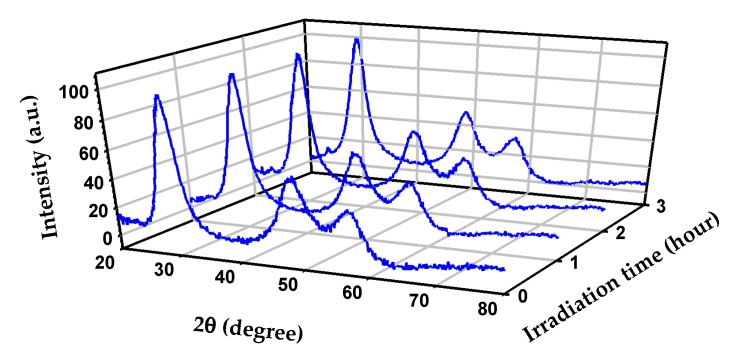
Powder XRD patterns for ZnS before (0 h) and after 1, 2, and 3 h of irradiation. Further experimental details from this illustrative figure can be found in references [2,9].

Figure 4 exemplifies the use of XRD to show that the same ZnS colloids from Figure 2 remained stable during several hours of irradiation [2,9]. The same stable behavior was recorded by XRD even after a second round of photocatalytic processing as well as by TEM–EDS and Raman analyses [2,9]. These complementary techniques indicated that for the photocatalyst to remain stable, a substitution mechanism consuming the sulfide hole scavenger protected the integrity of ZnS during irradiation [2]. Furthermore, there are examples in the literature showing typical demonstrations of the reusability of the photocatalyst and reproducibility of the data, such as that provided by Aguirre et al. for a Cu_2_O/TiO_2_ nanocomposite. In the previous work, Aguirre et al. repeated four cycles of irradiation of Cu_2_O/TiO_2_ lasting 1 hour and concluded that the production of carbon monoxide from the reduction of CO_2_ in the presence of water vapor as a hole scavenger was reproducible [21].

## 5. Proposing a Meaningful Mechanism

**Figure 5:**
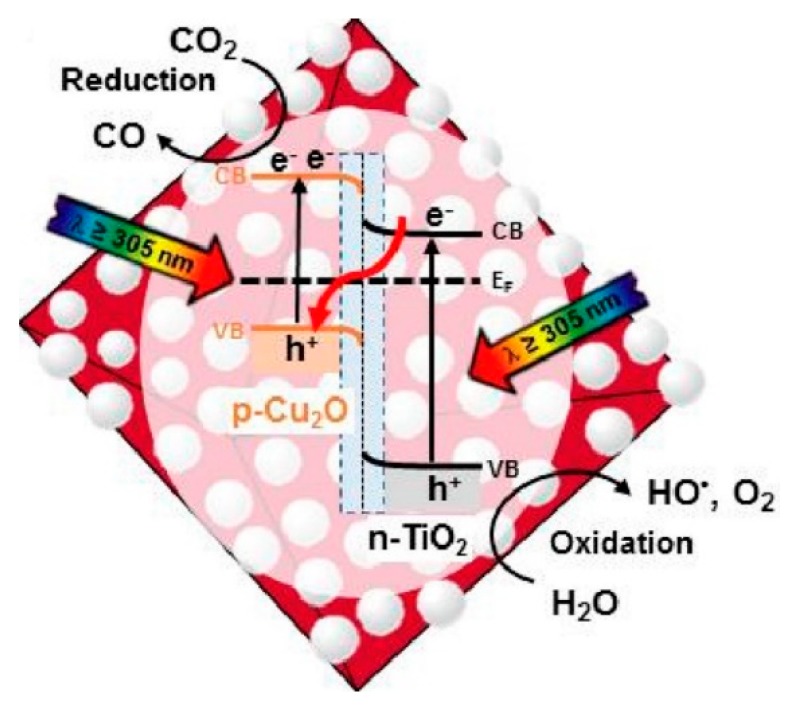
Proposed operative mechanism for the reduction of CO_2_(*g*) into CO(*g*) during UV–visible irradiation at *λ* ≥ 305 nm of an octahedral Cu_2_O/TiO_2_ nanocomposite in the presence of H_2_O(*g*). Adapted with permission from reference [21].

A final consideration for a praiseworthy work with photocatalytic systems is to include a meaningful mechanistic proposal that integrates all observations. Such a proposal must include both common phenomena of reduction and oxidation reactions that occur simultaneously in photocatalysis (i.e., CO_2_ reduction and water splitting). Therefore, the analysis of reactant decay and/or product growth from both reduction and oxidation reactions must be covered. Often, the yield of products is only monitored from the reduction reactions (i.e., CO, CH_4_, and/or CH_3_OH production during CO_2_ reduction, and H_2_ generation in water reduction), disregarding completely the oxidation reactions [41]. Figure 5 shows an example for the proposed reaction mechanism of CO_2_ reduction on the surface of a Cu_2_O/TiO_2_ nanocomposite in the presence of water vapor as the hole scavenger [21]. Figure 5 was supported by characterizing the processes occurring at both the conduction and valence bands of the heterostructure operating a z-scheme mechanism of charge transfer [21]. In order to improve the photocatalytic efficiency of the reduction process, it is a common practice to add some sacrificial electron donors (SEDs) during the reaction. The addition of SEDs enhances the selectivity and efficiency of the reduction cycle by scavenging the holes and reducing the rate of charge carrier recombination [42]. Although the addition of such SEDs helps to understand the pathway followed during electron transfer, it does not accurately describe the yield of the reduction process.

Several organic molecules such as alcohols, organic acids, and hydrocarbons and inorganic ions such as sulfide (S^2−^) and sulfite (SO_3_^2−^) in aqueous phase are usually employed as SEDs in different photocatalytic systems [41,42]. Organic molecules can produce C_1_ products, hydrogen, etc. via oxidation reactions, and inorganic ions after protonation can also undergo oxidation reactions to produce hydrogen [41]. The associated problem is that often the oxidation products of SEDs are considered as reduction products. Therefore, it is necessary to determine the contribution of SEDs to the overall product in the photocatalytic reaction and provide a step-by-step mechanistic pathway of both reduction and oxidation reactions. For the specific case of work reporting the degradation rate of a dye as a photocatalytic test easily monitored by UV–visible spectroscopy, this sole work is not sufficient for reporting photocatalytic activity. A complex matter is that dyes (i.e., methylene blue, rhodamine B, etc.) can undergo irreversible transformation under visible light irradiation via different mechanisms, such as hydroxyl radical-induced oxidation, photosensitized degradation, singlet generation, etc. [43,44]. Therefore, efforts to explain the degradation mechanism of the dye (in addition to its loss rate) must be made for demonstrating photocatalytic activity for such systems. For this reason, the use of tests with dyes has been discouraged in favor of employing standards such as phenol to compare photocatalytic activities [15].
